# Tyrosine-containing peptides are precursors of tyramine produced by *Lactobacillus plantarum* strain IR BL0076 isolated from wine

**DOI:** 10.1186/1471-2180-12-199

**Published:** 2012-09-11

**Authors:** Maryse Bonnin-Jusserand, Cosette Grandvalet, Aurélie Rieu, Stéphanie Weidmann, Hervé Alexandre

**Affiliations:** 1 AgrosupDijon, Valmis UMR PAM, Université de Bourgogne, Institut Universitaire de la Vigne et du Vin Jules Guyot, rue Claude Ladrey, BP 27877, 21078, Dijon Cedex, France

**Keywords:** Tyramine, Peptides, *Lactobacillus plantarum*, Wine

## Abstract

**Background:**

Biogenic amines are molecules with allergenic properties. They are found in fermented products and are synthesized by lactic acid bacteria through the decarboxylation of amino acids present in the food matrix. The concentration of biogenic amines in fermented foodstuffs is influenced by many environmental factors, and in particular, biogenic amine accumulation depends on the quantity of available precursors. Enological practices which lead to an enrichment in nitrogen compounds therefore favor biogenic amine production in wine. Free amino acids are the only known precursors for the synthesis of biogenic amines, and no direct link has previously been demonstrated between the use of peptides by lactic acid bacteria and biogenic amine synthesis.

**Results:**

Here we demonstrate for the first time that a *Lactobacillus plantarum* strain isolated from a red wine can produce the biogenic amine tyramine from peptides containing tyrosine. In our conditions, most of the tyramine was produced during the late exponential growth phase, coinciding with the expression of the *tyrDC* and *tyrP* genes. The DNA sequences of *tyrDC* and *tyrP* in this strain share 98% identity with those in *Lactobacillus brevis* consistent with horizontal gene transfer from *L. brevis* to *L. plantarum*.

**Conclusion:**

Peptides amino acids are precursors of biogenic amines for *Lactobacillus plantarum* strain IR BL0076.

## Background

Biogenic amines (BA) are molecules found in a wide range of fermented foods and can present a health hazard, including food poisoning, following consumption [1,2]. The BA histamine and tyramine in particular cause hypertension and headaches [3]. BA in foods are mainly produced through the decarboxylation of amino acids (AA) by lactic acid bacteria (LAB) [4]. From a physiological point of view, BA production could help LAB to survive in acidic conditions by the production of metabolic energy [5]. Indeed the decarboxylation reaction from AA to BA, coupled to the transport, provides a proton motive force composed of a pH gradient (alkaline inside the cell) and a membrane electric potential (negative inside). This mechanism was described in *Lactobacillus buchneri* for histamine production by Molenaar et al. [6], and more recently in *Lactobacillus brevis* for tyramine conversion from tyrosine by Wolken et al. [7]. Histamine [8], putrescine [9], tyramine [10] and cadaverine [11] are the main BA found in wine and are produced, during malolactic fermentation and storage, by LAB of various genera, notably *Oenococcus*, *Lactobacillus*, *Leuconostoc* and *Pediococcus*. The main producers of tyramine are species from the *Lactobacillus* genus [10]. Usually genes responsible for BA production are organized in clusters and are carried on genetic mobile elements integrated *via* horizontal gene transfer [12]. This explained the variability observed between strains for BA accumulation. Tyramine-producing bacteria carry a *tyrDC* cluster composed of four genes: *tyrS* encoding a tyrosil-tRNA synthetase, *tyrDC* encoding a decarboxylase, *tyrP* the tyrosine/tyramine transporter and *nhaC* encoding an Na^+^/H^+^ antiporter. This genetic organization has been described through LAB including *Enterococcus faecalis*[13], *Lactococcus lactis*[14] and *Lactobacillus brevis*[15].

Several studies have investigated factors influencing BA production in wine. Low pH [8], high ethanol concentration and low concentrations of pyridoxal-5-phosphate [16] favor reductions of BA accumulation. The BA content of wine also varies between viticultural regions, grape varieties [4,17] and vintages [18]. To avoid BA accumulation, commercially selected malolactic starters are added [4,19] based on RAPD-PCR typing and selected for their technological performances to ensure MLF beginning and also wine quality [20]. One of the major factors affecting BA production is the concentration of amino acids or, more broadly, nitrogen compounds [1]. Free amino acids (AA) favor BA formation: the histidine and the tyrosine decarboxylases are both enzymes induced in *Lactobacillus* sp. by histidine [21] and in *Lactobacillus brevis* and *Lactobacillus hilgardii* by the addition of tyrosine [10]. The AA and biogenic amine contents of wine have been analyzed by HPLC to assess the relationships between the two classes of molecules [22,23]. When BA reached the detection threshold, a correlation was made between high amounts of AA and increased BA accumulation. Bach et al. [24] reported that the final concentration of BA increases if nitrogen compounds are added during alcoholic fermentation. Also, storage on lees [4] increases BA production due to the availability of nitrogen compounds released from yeasts undergoing autolysis. Yeast autolysis involves the breakdown of yeast cell membranes and the release of hydrolytic enzymes that then degrade components in the medium [25]; consequently, the medium is enriched in protein, peptides and free amino acids. Alexandre et al. [26] shown that yeasts can release until 40 mg.L^-1^ of peptides during autolysis. Furthermore wine peptides contain between 5 and 7 mg.L^-1^ of tyrosine [27] and contribute to the overall nitrogen compound [28]. So peptides, as well as free AA, could also be involved in BA production.

Moreover, LAB performing malolactic fermentation (MLF) express a proteolytic system; they therefore can degrade peptides in the extracellular or intracellular media and then decarboxylate AA to produce BA. Indeed, *O. oeni* exhibits a proteolytic activity against peptides in both white and red wines [29,30], and an extracellular protein, EprA, with protease activity has been characterized [31]. Nevertheless, it seems that the proteolytic activity of *O. oeni* is dependent on both the composition of the medium and the bacterial growth phase [32]. A proteinase named PrtP produced by one isolate of *Lactobacillus plantarum* has been identified [33]. The aim of this study was to test the ability of *L. plantarum* to produce tyramine from synthetic peptides containing tyrosine, and to investigate whether peptides are hydrolyzed either inside the cell or in the extracellular medium.

Different sorts of synthetic peptides, containing two to four amino acids, were used to conduct these experiments depending on either the size or the place of the tyrosine residue. It is well known that transporters and intracellular peptidases have preferences for peptide size (for both). Indeed, various types of peptide transport have been described in the model LAB *Lactococcus lactis*. It harbors a well-characterized Opp transport system, of the ABC transporter family, which can transport peptides containing 4 to 35 residues [34]. The proteins DtpT and DppP are specialized in the transport of dipeptides [35] and tripeptides [36], respectively. *L. plantarum* has also an essential system for peptides uptake [37].

Peptidases display specificities for the position of residues in peptides. Many bacteria express specific intracellular peptidases able to hydrolyze peptide bonds. For example, the PepN aminopeptidase, has been described in a wide range of LAB including *Lactobacillus helveticus*[38], *Lactobacillus delbrueckii*[39] and *Lactococcus lactis*[40], and hydrolyze the residue located at the N-terminus of peptides. Di- and tri-peptidases, such as PepV, isolated from *Lactococcus lactis*[41] and several lactobacilli, are able to breakdown dipeptides containing a Gly redisue at the N-terminus. In this study two of the peptides used (Gly-Leu-Tyr and Gly-Gly-Tyr-Arg) have a Gly residue at the N-terminus. Growth, tyramine production and expression of *tyrDC* and *tyrP* were also investigated in media with either free tyrosine or a mix of selected synthetic peptides.

## Results and discussion

*Lactobacillus plantarum* is frequently isolated from red wine undergoing malolactic fermentation (MFL) and it usually contributes to production of tyramine [42]. It is auxotrophic for tyrosine and thus is suitable for studying the production of tyramine from peptides containing tyrosine.

### The tyrDC and tyrP genes of *L. plantarum* IR BL0076

Based on 16S RNA gene sequencing [GenBank : JX025073] and multiplex PCR using *recA* gene-derived primers [43] (data not shown), a lactic acid bacterial strain isolated from wine and able to produce tyramine was identified as *L. plantarum*, and was named IR BL0076. To characterize the *tdc* pathway of this strain, we amplified and sequenced the region carrying *tyrDC* and *tyrP*; the complete sequences of the *tyrDC* and *tyrP* genes in *Lactobacillus plantarum* have not previously been reported although *tyrDC* was partially sequenced by Arena et al. [42]. The presence of the *tyrDC* gene is strain-specific, and sequenced *L. plantarum* genomes, like those of strains WCFS1 and ATCC 14917, do not carry the genes of the *tyrDC* pathway.

**Figure 1:**
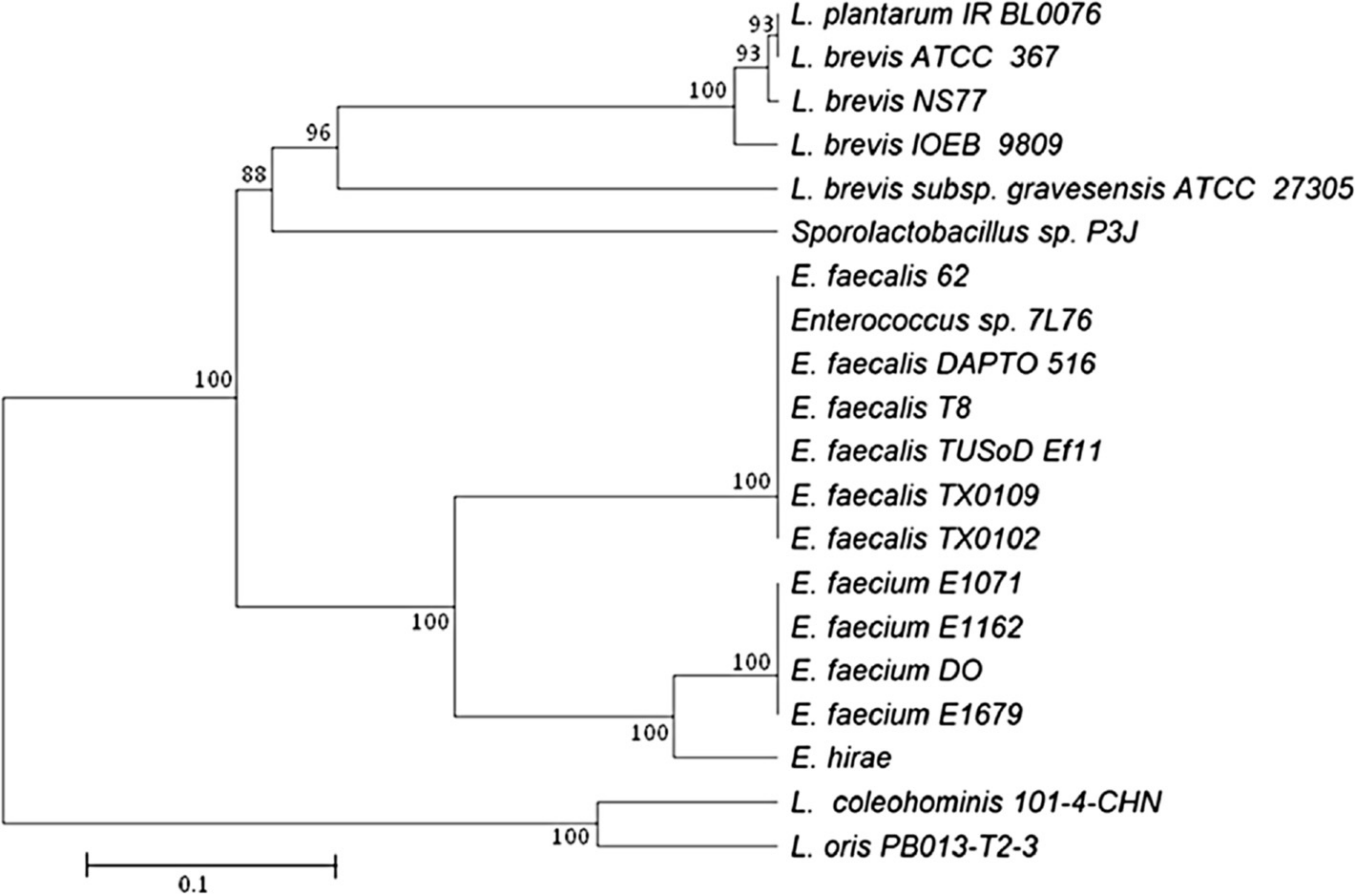
**Phylogenetic tree comparing 21 TyrDC sequences from various *Lactobacillus *strains. **The amino acid sequences were aligned using the multiple alignment program CLUSTAL W2. The phylogenetic tree was constructed by using the TreeTop from the GeneBee. Bootstrap values are expressed in percentages and indicated at nodes. The amino acid sequences of TyrDC were obtained from the following accession numbers entries: [GenBank : AF446085] (*L. brevis* IOEB 9809), [GenBank : YP_796294.1] (*L. brevis* ATCC 367), [GenBank : ABY71221.1] (*L. brevis* NS77), [GenBank : ZP_03940842.1] (*L. brevis* subsp. *gravesensis* ATCC 27305), [GenBank :AEB91325.1] (*Sporolactobacillus* sp. P3J), [GenBank : AAQ73505.1] (*E. hirae*), [GenBank : ZP_05553037] (*L. coleohominis* 101-4-CHN), [GenBank : ZP_07729457] (*L. oris* PBo13-T2-3), [GenBank :ZP_06679761] (*E. faecium* E1071), [GenBank : ZP_06677337] (*E. faecium* E1162), [GenBank : ZP_00602894.1] (*E. faecium* DO), [GenBank : ZP_06698865.1] (*E. faecium* E1679), [GenBank : CAF33980] (*E. durans* IPLA 655), [GenBank : ZP_05559869] (*E. faecalis* T8), [GenBank : ZP_07768147] (*E. faecalis* DAPTO 516), [GenBank : ZP_07771864] (*E. faecalis* TX0102), [GenBank : ZP_07569615] (*E. faecalis* TX0109), [GenBank : CBL32775] (*Enterococcus* sp. 7 L76), [GenBank : ADX79254] (*E. faecalis* 62) and [GenBank : ZP_04646316] (*E. faecalis* TUSoD Ef11).

Primers tyrSa and nhaCa were used to amplify the *tyrDC* and *tyrP* genes from *L. plantarum* IR BL0076; a fragment of the expected size (3.8 kbp) was obtained and sequenced. The DNA sequence [GenBank : JQ040309] shares 98% identity with those of the *L. brevis* NS77 *tyrDC* and *tyrP* genes. The deduced amino acid sequence showed 99 to 100% identity with TyrDC and TyrP from *L. brevis* NS77, IOEB 9809 and ATCC 367 strains (see Additional file 1). Regarding this alignment, the TyrDC sequence from *L. brevis* NS77 showed six amino acids substitutions compared to the three other strains: A63, N112, P184, S276, A564 and V572 are changed in E63, S112, Q184, R276, V564 and A572 respectively. Moreover the amino acid A564 is also changed in V564 for *L. brevis* ATCC 367. Lower identity was obtained with TyrDC from *Lactobacillus brevis* subsp. *gravesensis* (76%). Identity with the sequences in other lactobacilli, such as *Sporolactobacillus* sp. *Enterococcus hirae, Enterococcus faecium, Enterococcus durans* and *Enterococcus faecalis* ranges between 66 and 80%. Amino acid sequence similarity with the sequences from *Lactobacillus coleohominis* 101-4-CHN and *Lactobacillus oris* strains is lower, between 23 and 60%, with better conservation for the TyrDC (60% for both) than TyrP (55 and 54% respectively) amino acid sequences. A phylogenetic tree was constructed to investigate the evolutionary relationships between these proteins. Based on the sequence divergence in amino acid TyrDC sequences (Figure 1), the phylogenetic tree reveals that *L. plantarum* TyrDC is closely related to those of *L. brevis* proteins and made one cluster clearly separated. Similar results were obtained when phylogenetic tree was constructed with TyrP amino acid sequences (data not shown). These results confirm that the organization of this *L. plantarum tdc* locus is similar to those described for other LAB strains, with contiguous *tyrDC* and *tyrP* genes. The phylogenetic tree analysis is consistent with the *tdc* locus of *L. plantarum* IR BL0076 strain having been transferred horizontally from *L. brevis*.

### Growth of *L. plantarum* with peptides containing tyrosine

Peptides of different sizes were used: a dipeptide Tyr-Ala containing the tyrosine residue at the N-terminus, a tripeptide Gly-Leu-Tyr with the tyrosine at the C-terminus, and a peptide of four amino acids Gly-Gly-Tyr-Arg, where the tyrosine is in an internal position.

**Figure 2:**
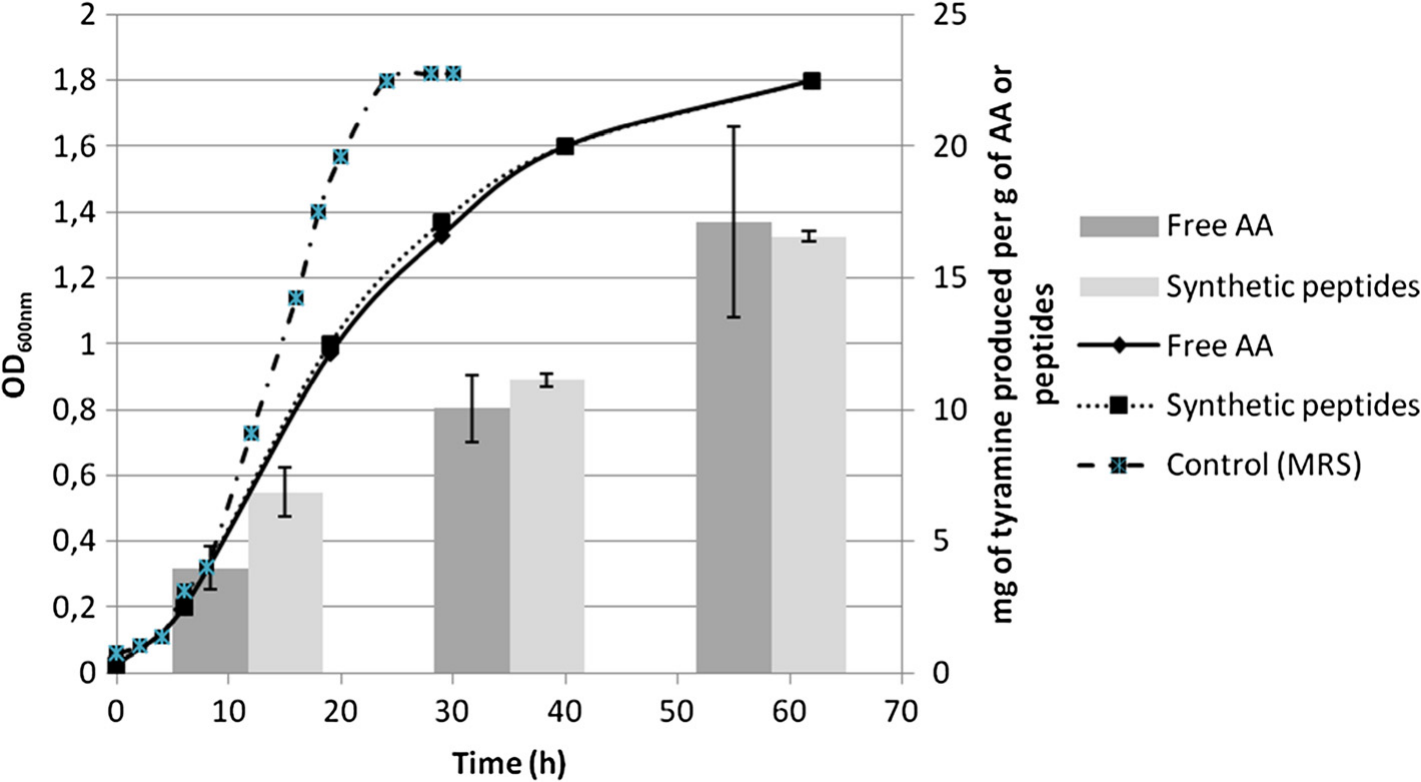
**Influence of tyrosine or tyrosine containing peptides on growth and tyramine production by *Lactobacillus plantarum *IR BL0076. ***Lactobacillus plantarum *IR BL0076 was grown in MRS medium (control curve; dashed line), synthetic medium with free tyrosine (continuous line) or in medium containing synthetic peptides as the sole tyrosine sources (dotted line). Tyramine was assayed by HPLC after various times of growth of *L. plantarum* IR BL0076 (OD_600nm_ = 1.0; 1.6; 1.8), in both culture media. Each value is the mean ± SD of three independent experiments.

The growth was monitored by measuring the OD at 600 nm. *L. plantarum* IR BL0076 was able to grow in the synthetic medium either with free amino acids (medium 1) or synthetic peptides containing tyrosine (medium 2). The growth curve was the same in the two media (Figure 2), but not in MRS medium (control). Indeed at 28 h of growth, the maximum OD at 600 nm of 1,8 was reached in MRS rich medium, while in the synthetic poor media, the OD at 600 nm was 1,3. *L. plantarum* is auxotrophic for L-tyrosine [44], and indeed *L. plantarum* IR BL0076 could not grow in the synthetic medium used in this study without the inclusion of tyrosine. Therefore, the synthetic peptides in medium 2 were presumably metabolized even during the early stages of culture to release tyrosine and to allow the growth. This is consistent with the demonstration that two *Lactobacillus* strains (*Lactobacillus homohiochii* and *Lactobacillus curvatus*) isolated from sausages, express tyrosine and ornithine decarboxylase activities allowing growth at early stages of culture [45]; both strains display extracellular proteolytic activity which reaches a maximum in the early exponential growth. This activity is higher when the cells were grown in a peptide-rich medium. However, peptide transport and a subsequent intracellular hydrolysis is also plausible. Although LAB proteinases have a broad specificity and release oligopeptides in the range of 4 to 8 AA, intracellular peptidases are required for the complete degradation of peptides [46].

### Tyramine production by *lactobacillus plantarum* IR BL0076

Supernatant harvested from the cultures after various times of growth was analyzed by HPLC to determine tyramine production (Figure 2). From Gomez-Alonso et al. [47], the detection limit for aminoenone derivative of tyramine is 0.02 mg.L^-1^. Tyramine was identified by HPLC-MS (Table 1). At culture OD_600nm_ = 0.2, no tyramine was detected in any culture. Tyramine was detected, at similar concentrations, in cultures in both media from OD_600nm_ = 1.0. Concentrations of tyramine for both media were measured between 1.6 and 5.1 mg.L^-1^ (minimal and maximal measures respectively). The concentrations measured in both media are usually found in wine. Indeed in red wines, tyramine concentration can reached 28 mg.L^-1^ which is the upper limit, but most of time these concentrations are lower than 2.5 mg.L^-1^[48]. Therefore, *L. plantarum* was able to synthesize tyramine similarly from free tyrosine and from peptides containing tyrosine.

**Table 1:** Identification of tyrosine and tyramine by HPLC-MS

**Amine**	**Derivated mass**	**Molecular ion**	**Caracteristic ions**
Tyramine	307	306	306,260,214,186
Tyrosine	351	350	350, 306, 260

Tyramine was produced throughout growth and it accumulated as the biomass increased. This is consistent with the biological role of tyramine: it is involved in energy production through decarboxylation coupled to transport [5]. This is the first demonstration that tyramine can be produced from peptides containing tyrosine and therefore that free tyrosine is not the only precursor for tyramine production. We studied the expression of the *tyrDC* and *tyrP* genes to determine whether it was growth phase-dependent and/or nitrogen source dependent.

### tyrDC and tyrP expression

The *tyrDC* and *tyrP* genes are co-transcribed in *E. faecalis*[13], *L. brevis*[15] and *Sporolactobacillus* sp. [49]. A complete transcriptional analysis of the four genes of the operon was made in *Lactobacillus brevis* IOEB 9809 [15]. Even if *tyrDCtyrP* transcripts were the most abundant, other polycistronic mRNA were described as: *tyrS-tyrDC-tyrP-nhaC* and *tyrS-tyrDC*, as well as *tyrP-nhaC.* So *tyrDC* and *tyrP* can be transcribed from different manner. *L. plantarum* IR BL0076 *tdc* locus sequences was analysed using ARNold, an interface allowing localization of Rho-independent terminators in any bacterial sequence. (na.igmors.u-psud.fr/toolbox/arnold/). A predicted transcription terminator (−11.70 kcal/mol) localized at the 3′ end of TyrP coding region was identified. Erpin and RNAmotif programm predict the 5′ end position of this predicted transcription terminator at the nucleotide 3402 of the locus.

To check the presence of a bicistronic *tyrDC-tyrP* in the IR BL0076 isolate, we used Reverse-Transcription-PCR experiments and primers tdcf and tyrPLpR located inside the *tyrDC* and *tyrP* genes respectively to study their expression in *L. plantarum*. An amplicon of 1,761 bp was obtained using cDNA obtained from RNA extracted from cultures on each medium 1 and medium 2 as the template. The length of the RT-PCR product indicates that *tyrP* is part of a polycistronic mRNA including *tyrDC*. As the four genes of the tyrosine decarboxylase operon are part of a genetic island, as described for *L. brevis*[12], they have been disseminated through lactic acid bacteria *via* a horizontal gene transfer [49]. So it is expected that they are regulated in the same way in all enterococci and lactobacilli including *L. plantarum*.

**Figure 3:**
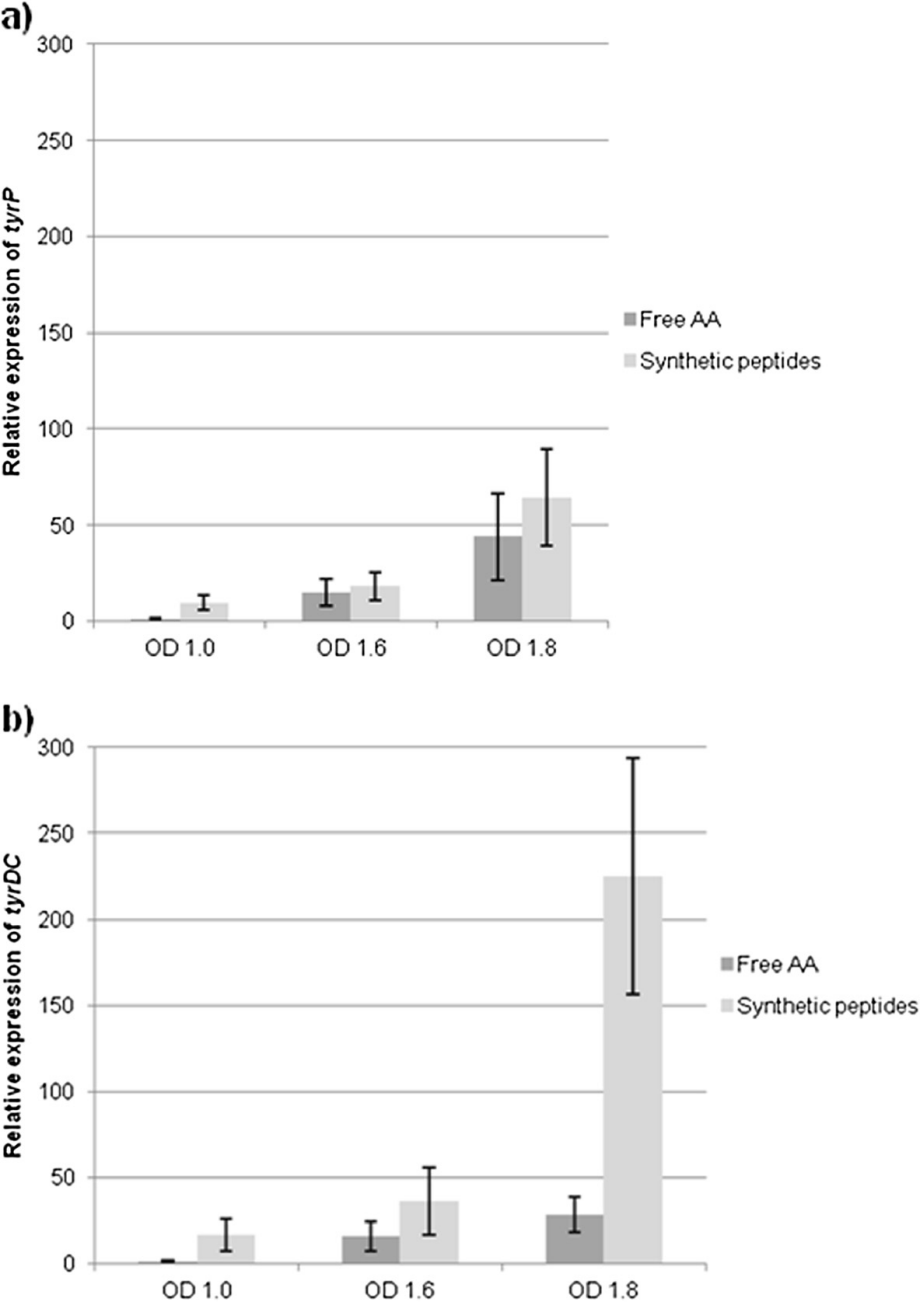
**Relative expression of: a) the tyramine transporter *tyrP *and b) the tyrosine decarboxylase *tyrDC *in *L. plantarum *IR BL0076 grown with free tyrosine or tyrosine-containing peptides. ** Expression was measured at three different OD_600nm_. Each value is the mean + − SD of three independent experiments. The difference between the values labeled a are significantly different, likewise those labeled b (ANOVA, p < 0.05). Significant differences between Free AA (medium 1) and Synthetic peptides (medium 2) media for each OD are indicated with an asterix.

To study the tyrosine transport, expression *tyrP* and *tyrDC* was similarly analyzed by RT-qPCR. The expression of *tyrP* increased during growth in both medium 1 and medium 2, with a maximum at OD_600nm_ = 1.8 (Figure 3a), and was significantly stronger during the stationary phase than during early exponential growth. The expression of *tyrP* paralleled the accumulation of tyramine in both media (Figure 1). This is coherent with what has been found for other bacteria producing biogenic amines, for example *Streptococcus thermophilus*[50], which produces histamine at the end of its growth, with an increase in the expression of the decarboxylase *hdcA*. The expression profile of *tyrDC* during growth was very similar to that of *tyrP* (Figure 3b). Both *tyrDC* and *tyrP* were significantly more strongly expressed during the early exponential growth phase in peptide medium (medium 2) than tyrosine medium (medium 1). Also, the expression of *tyrDC* during the stationary growth phase was higher in peptide than tyrosine medium. This higher expression in peptide medium was not associated with a higher concentration of tyramine, and its physiological significance is not clear. This is the first study of the influence of peptides on *tyrDC* and *tyrP* expression in LAB.

### Proteolysis of peptides

**Figure 4:**
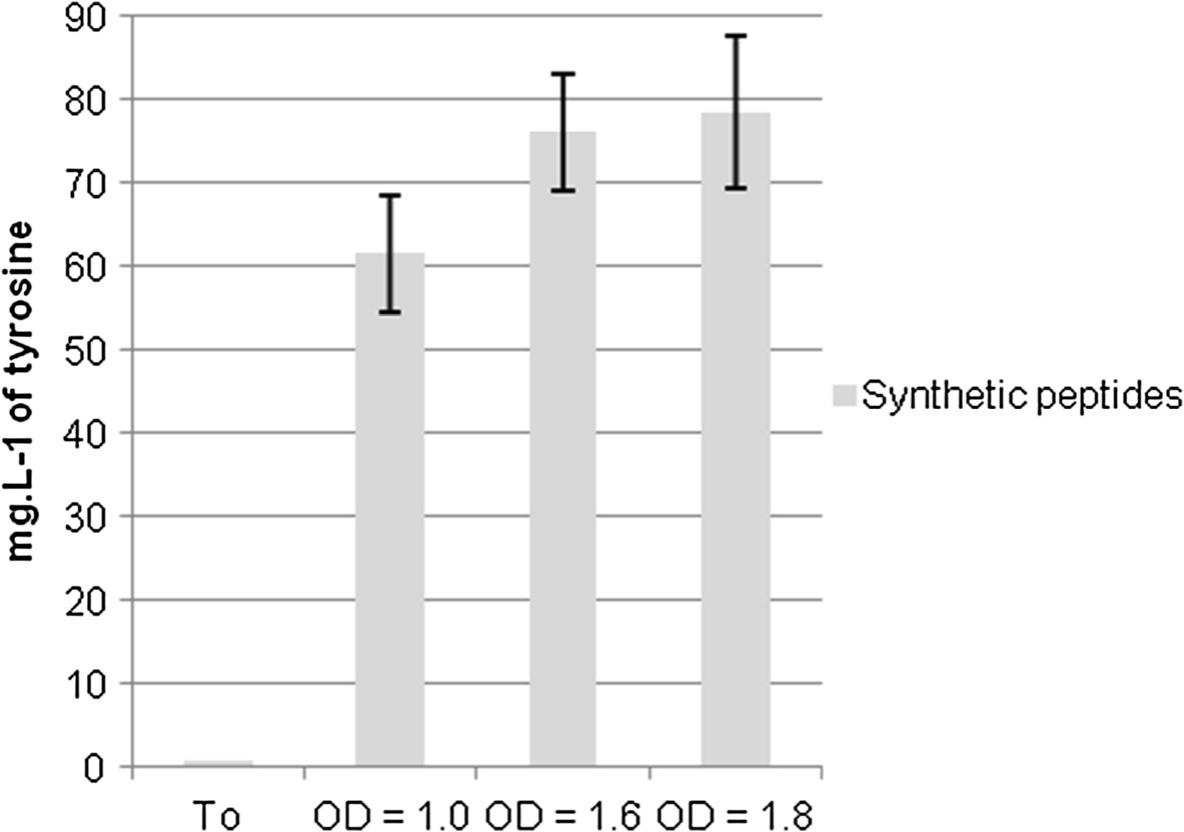
**Tyrosine concentration in the supernatants of the culture media containing synthetic peptides.** To corresponds to the tyrosine concentration in the medium before inoculation with *L. plantarum* IR BL0076. Each value is the mean ± SD of three independent experiments.

Tyramine could be produced from peptides in two ways. Peptides could be hydrolyzed in the extracellular medium by proteinase(s). Alternatively, they could be transported inside the cell by a peptide transporter, then hydrolyzed by intracellular peptidases, and the released tyrosine decarboxylated to give tyramine which could be exported by the TyrP permease. However, this second possibility is unlikely, because the TyrP transporter catalyses the exchange of tyrosine and tyramine. We assayed tyrosine in the culture medium during the growth of *L. plantarum* to determine whether peptides were hydrolyzed extracellularly (Figure 4).

In the peptide medium 2, the concentration of tyrosine was measured when the cultures reached the exponential growth phase. Therefore synthetic peptides were, as expected, hydrolyzed in the extracellular medium. Tyramine is presumably produced from the hydrolysis of peptides throughout the growth of the culture. The genome of the sequenced strain, *L. plantarum* WCFS1, contains genes encoding uptake systems for peptides, and in particular the oligopeptide transport system Opp. Once internalized, peptides can be degraded by peptidases. *L. plantarum* WCFS1 has nineteen genes encoding intracellular peptidases with diverse specificities [37]. Note also that one isolate of *L. plantarum* produces an extracellular proteinase, PrtP [33], and proteolytically active strains produce one or more other extracellular proteinase(s). Our experiments do not exclude the possibility that peptides are also imported and hydrolyzed inside the cell. Indeed, tyrosine generated by extracellular proteinase(s) could be exchanged with tyramine that has been formed inside the cell after decarboxylation of tyrosine derived from intracellular hydrolysis of peptides. However, no such mechanism has ever been described in any lactic acid bacteria.

## Conclusion

This is the first demonstration that peptides containing amino acids precursors of biogenic amines (BA) can be used by bacteria to produce such BA. We show that peptides are, in fact, broken down into amino-acids (AA), which are the BA precursors in the extracellular medium. Peptide transport has a high energy cost for the cell and requires the hydrolysis of ATP [46]. This degradation of peptides outside the cell is thus a simple and energetically favorable way to obtain free AA for metabolic needs.

This study is of technological interest, because most enological practices aim at enriching wine in nutrients to enhance the performance of yeasts and lactic acid bacteria, and to improve wine quality. This is why the influence of nitrogen sources on biogenic amines production has been extensively studied. Indeed, the presence of fine yeasts lees increase BA production, because of the wide range of nitrogen-containing precursors released [4]. Because nitrogen, and especially yeast-assimilable nitrogen, is the limiting factor for yeast development, musts are sometimes supplemented with nitrogen sources [24,51]. Thus, nutritive supplements, for example yeast autolysates containing amino acids and proteins, are added to must to activate alcoholic fermentation. It has been shown that after malolactic fermentation, the concentration of biogenic amines is higher in wine produced with supplemented than unsupplemented must [52]. Therefore, as LAB are able to produce biogenic amines both from amino acids and directly from peptides, enological practices favoring the development of alcoholic fermentation and malolactic fermentation have to be carefully monitored.

## Methods

### Bacterial strain and growth conditions

*Lactobacillus plantarum* IR BL0076 (provided by Inter-Rhône, France) was isolated from wines of the Rhône Valley during aging. This strain produces tyramine.

### Study of the tdc pathway of *L. plantarum*

Primers tyrSa and nhaCa (Table 2) were used to sequence the *tyrDC* and *tyrP* genes. These primers were designed according to the sequence of the *tdc* locus of *L. brevis* (accession number [GenBank: EU195891]).

**Table 2:** Oligonucleotides used in this study

**Primer name**	**Gene function**	**Primer sequence**	**Product size (bp)**	**Source**
tyrSa	tyrosil-tRNA synthetase	GTACGGATACGGACGCACAA	3815	This work
nhaCa	antiporter Na+/H+	CCTAGTGAAAAATGGACAGC		
tdcf	tyrosine decarboxylase	CAAATGGAAGAAGAAGTTGG	1761	[55]
tyrPLpR	tyrosine/tyramine transporter	TAGTTCCCAACTCACCAGAAA		This work
tdcBF	tyrosine decarboxylase	GCCTTAGAAAGTATTATTCG	118	This work
tdcBR		AGCGACAATCTTATCAATGC		
tyrPLpF	tyrosine/tyramine transporter	TATGATTGCCACCGTTCGTTC	128	This work
tyrPLpR		TAGTTCCCAACTCACCAGAAA		
*ldhD* (Forward primer)	dehydrogenase	ATCGGTACTGGTCGGATTGG	123	[56]
*ldhD* (Reverse primer)		GGTGTCAACGTACATGCCTTC		
*gyrA* (Forward primer)	gyrase	GTTCGTCTCATGCGGTTAGG	85	[56]
*gyrA* (Reverse primer)		AACTGGTGCCTCAGTCGTTG		

*L. plantarum* IR BL0076 was inoculated at OD_600nm_ = 0.025 in a nitrogen-free synthetic medium containing the following components: 5 g.L^-1^ glucose, 3.5 g.L^-1^ fructose, 10 g.L^-1^ D,L- malic acid, 0.6 g.L^-1^ KH_2_PO_4_, 0.45 g.L^-1^ KCl, 0.13 g.L^-1^ CaCl_2_, 2H_2_O, 0.13 g.L^-1^ MgSO_4_, 7H_2_O, 3 mg.L^-1^ MnSO_4_, H_2_O, and 1 mL.L^-1^ Tween 80, at pH 5.

Amino acids were added one by one as nitrogen sources according to Terrade et al. [53]. This medium corresponds to the first culture condition where amino acids are free and contains 1.6 mM of tyrosine. Otherwise, in a second condition, tyrosine was replaced by 1.6 mM of a mix of synthetic peptides containing tyrosine: Gly-Gly-Tyr-Arg, Tyr-Ala and Gly-Leu-Tyr purchased from Sigma-Aldrich (Saint Quentin Fallavier, France). Aliquots of 50 mL of culture were harvested after various times of the growth and centrifuged for 10 min at 6,000 rpm. The pellets were stocked at −20°C until RNA extraction. A 1 mL sample of each supernatant was derivatized and analyzed by HPLC to assay biogenic amines and amino acids. The rest of the supernatant was stored at −20°C.

### Amino acid and biogenic amine analysis by HPLC

Free AA and BA were analyzed by HPLC using the method described by Gomez-Alonso et al. [47]. The derivatization reaction was performed by adding 1.75 mL of borate buffer pH 9, 1 mL of methanol, 40 μL of internal standard (2,4,6-trimethylphenethylamine hydrochloride, 2 mg.mL^-1^), and 30 μL of DEEMM (diethyl ethoxymethylenemalonate) to 1 mL of target sample. The samples were placed for 30 min in an ultrasound bath, then heated to 70°C for 2 h to allow complete degradation of excess DEEMM and reagent byproducts. The analyses were performed on a Varian HPLC (Varian Inc., Walnut Creek, CA) using an Alltech (Grace, Templemars, France) HPLC column (C18-HL), particle size 5 μm (250 mm × 4.6 mm), maintained at 16°C, with a binary gradient. Phase A was modified with 10 mM ammonium acetate pH 5.8 to allow the identification of AA and BA by mass spectrometry. The mobile phase, phase B, was 80:20 mixture of acetonitrile and methanol and the flow rate a constant 0.9 mL.min^-1^.

### HPLC-MS conditions

LC-MS/MS analyses were performed on a ThermoFinnigan TSQ Quantum triple quadrupole mass spectrometer equipped with a standard electrospray ionization source fitted with a 100 μm i.d. H-ESI needle. HPLC was performed using an Accela™ LC pump from ThermoFinnigan (San Jose, CA, USA) equipped with an Accela autosampler (for HPLC conditions, see paragraph above). The flow from LC was split using an analytical fixed flow splitter (split ratio = 1:10, post-column) from Analytical Scientific Instruments (El Sobrante, CA, USA). The data were processed using Xcalibur software (ThermoFinnigan).

The source spray head was oriented at an angle of 90°C orthogonal to the ion-transfer tube. The mass spectrometer was operated in the negative ion mode in the range of *m/z* 90–900 with a scan time of 1 s. Nitrogen was used as the sheath gas, ion sweep gas and the auxiliary gas, at 30, 5 and 30 (arbitrary units), respectively. The spray voltage was 3 kV, the tube lens offset −132 V and the skimmer offset 5 V. The ion transfer capillary temperature and vaporizer temperature were 250 and 300°C respectively. All the amines give a *m*/*z* signals that correspond to the structure [*M*-H]^-^.

Each amine was injected at 1 to 10 μg.mL^-1^ for mass characterization. Collision-induced dissociation (CID) was performed from 20 to 30 eV under 1.5 mTor of argon.

### PCR amplification

*L. plantarum* identification was performed by 16S ribosomal RNA gene sequencing and multiplex PCR using *recA* gene-derived primers [43]. Chromosomal DNA from *L. plantarum* was extracted using the Wizard Genomic Kit (Promega, Charbonnières les Bains, France). Amplification and sequencing of the 16S gene was performed using the High Fidelity Taq polymerase (Roche, Meylan, France) and the universal primers BSF8 and BSR1541 [54]. Amplification conditions were 94°C for 2 min, 10 cycles of 94°C for 15 s, 52°C for 30 s, and 72°C for 1 min 30 s, followed by 20 cycles with an additional time of 5 s for each elongation reaction and a final extension at 72°C for 10 min. Multiplex PCR protocol developed by Torriani et al. [43] was performed with Go Taq polymerase (Promega, Charbonnières les Bains, France) and was modified for dNTP concentration (0.2 mM inside of 12 μM) and for annealing time (20 s inside of 10 s). The *L. plantarum* IR BL0076 *tyrDC* and *tyrP* genes were amplified by PCR using High Fidelity Taq polymerase (Roche, Meylan, France) and primers tyrSa and nhaCa based on the *tyrS* and *nhaC* sequences which flanked *tyrDC* and *tyrP* genes of *L. brevis* NS77 [GenBank : EU195891]. Amplification was performed in a final volume of 50 μL, with 5 μL of Expand High Fidelity buffer (Roche, Meylan, France), 1 μL of 10 mM dNTP mix (Fermentas, Villebon sur Yvette, France), 1 μL of each primer at 20 μM, 2.6 U of Expand High Fidelity enzyme mix (Roche, Meylan, France), and 1 μL of extract DNA. The amplification program was applied in a Bio-Rad thermocycler following the manufacturer’s instructions for long fragments. PCR fragments were purified using the GenElute PCR purification kit (Sigma, Saint Quentin Fallavier, France) and sent to Benckman Coulter Genomics (United kingdom) for sequencing.

### Total RNA extraction and RT-PCR

*L. plantarum* RNA was extracted after various periods of growth in media 1 and 2 (when the cultures reached at OD_600nm_ = 1.1, 1.6 and 1.8). Aliquots of 25 mL of culture were harvested, and the cells pelleted and washed with 10 mL of Tris HCl 10 mM pH 8. The cells were then broken in 1 mL of Tri-Reagent (Sigma, Saint Quentin Fallavier, France) in a screw cap tube containing 200 mg of beads (100 μm) in a Precellys 24 ultrasound device (Ozyme, Saint Quentin en Yvelines, France) programmed as follows: 6500, 3 × 30 s, twice. Cell fragments were pelleted by centrifugation (12,000 × *g*, 10 min, 4°C) and the supernatant was transferred to an Eppendorf tube and 200 μL of chloroform was added. The tubes were then vortexed for 15 s and incubated at room temperature for 15 min. The samples were centrifuged at 12,000 × *g* for 15 min at 4°C. The upper layer was transferred to a new Eppendorf tube and 500 μL of isopropanol was added. The samples were mixed gently, incubated at room temperature for 15 min and centrifued at 12,000 × *g* for 10 min at 4°C. The supernatant was removed and the pellet was washed with 75% ethanol. The tubes were centrifuged at 12,000 × *g* for 10 min at 4°C and the resulting RNA pellets were dried and resuspended in 30 μL of RNase-free water (Fermentas, Villebon sur Yvette, France). These RNA samples were then purified with the RNeasy MiniKit (Qiagen, Courtaboeuf, France) and checked for yield and quality by measuring the OD ratio at 260, 280 and 320 nm in a BioPhotometer (Eppendorf, Le Pecq, France).

Aliquots of 2 μg of RNA were treated with 1 U of DNase I (Fermentas, Villebon sur Yvette, France) to eliminate residual DNA and used for PCR. A control PCR with irrelevant primers BSF8 and BSR1541 was carried out with the RNA to check the absence of any amplification. Total cDNA was then synthesized with iScript cDNA Synthesis Kit (BioRad, Marnes la Coquette, France) following the manufacturer’s recommendations. RT-PCR experiments were performed on cDNA with primers tdcf [55] and tyrPLpR (Table 2), and High Fidelity Taq polymerase (Roche, Meylan, France).

### Quantification of gene expression by real time quantitative PCR

Reverse transcription-quantitative real-time PCR (RT q-PCR), with iQ SYBR green supermix (BioRad, Marnes la Coquette, France) and the BioRad CFX96 Real-Time System was used for gene expression analysis.

First, primer specificity and efficacy were checked by using 10-fold serial dilutions of *L. plantarum* IR BL0076 DNA. The melting curves obtained showed the absence of primer dimers, and the calibration curves, for each pair of primers, showed a slope between 86.8% and 96.2%, and a regression coefficient between 0.997 and 1. Total cDNA was serially diluted (1 in 4), and a 5 μL aliquot was added to each well containing 20 μL of a mix of 12.5 μL of SYBR green supermix, 1 μL of each primer at 7 pmol. μL^-1^ and 5.5 μL of RNase-free water. The specific primers used to amplify particular cDNA sequences are given in Table 2. *ldhD* and *gyrA* are housekeeping genes used to normalize RNA expression data. These genes were used by Duary et al. [56] as the most stably expressed genes for RT-qPCR experiments in *L. plantarum*. Moreover *ldhD* gene was validated in *L. plantarum* for RT-qPCR experiments by Fiocco et al. [57]. Each run included a negative control with 5 μL of RNase-free water instead of cDNA, and a positive control using *L. plantarum* IR BL0076 DNA. The amplification program was as follows: 98°C, 30 s and 40 cycles of 95°C, 10 s; 60°C, 30 s. For each experiment, the condition “culture medium 1 (with free tyrosine), OD_600nm_ = 1.0” was used to calibrate the expression data. The analyses were performed on RNA extracted and purified from three independent cultures for each growth condition.

### Statistical analysis

R: A language and environment for statistical computing (R Development Core Team (2008); R Foundation for Statistical Computing, Vienna, Austria) was used for statistical analysis. Results were analyzed by one-way ANOVA and considered significant at p < 0.05.

### Sequence analysis and accession number

The 16S ribosomal gene sequence was analyzed using the Blast server for identification of Procaryotes ( http://bioinfo.unice.fr/blast/). Sequence similarity searches were carried out using Basic Local Aligment Search Tool (BLAST) on the JGI website ( http://www.jgi.doe.gov/). Multiple alignments were obtained using the CLUSTALW2 program on the EMBL-EBI web site ( http://www.ebi.ac.uk/). The tool TreeTop of GeneBee Molecular Biology Server was used for phylogenetic tree construction ( http://www.genebee.msu.su/genebee.html). The partial nucleotide sequence of the *tdc* locus and the 16S ribosomal DNA sequence *of L. plantarum* IR BL0076 are available in the GenBank database under the accession number [GenBank : JQ040309] and [GenBank : JX025073] respectively.

## Abbreviations

BA: Biogenic amines; AA: Amino acids; LAB: Lactic acid bacteria; RP-HPLC: Reverse-phase high-performance liquid chromatography; MLF: Malolactic fermentation; Rt-qPCR: Real time quantitative polymerase chain reaction; LC-MS/MS: Liquid chromatography-mass spectrometry/Mass spectrometry.

## Competing interests

This work was supported by the European Community’s Seventh Framework Program, grant agreement no. 211441-BIAMFOOD.

## Authors’ contributions

MB carried out all the analysis, and drafted the manuscript. CG participated in the design of the study, coordination and helped to draft the manuscript participated in the sequence analysis. AR and SW participated in the design of the study, especially the RT-QPCR experiments, coordination and helped to draft the manuscript. HA participated in the design of the study, coordinated all the work and helped to draft the manuscript. All authors read and approved the final manuscript.

## Supplementary Material

Additional file 1 **Sequence alignment of TyrDC from *L. brevis *and *L. plantarum*.** Click here for file
