# Supplementary material for: Tyrosine-containing peptides are precursors of tyramine produced by Lactobacillus plantarum strain IR BL0076 isolated from wine
Source: BMC Microbiol. 2012 Sep 11;12:199. doi: 10.1186/1471-2180-12-199 (PMC3492074; doi:10.1186/1471-2180-12-199)
Supplement: Additional file 1 — Sequence alignment of TyrDC from L. brevis and L. plantarum. [file 1471-2180-12-199-S1.doc]

Additional file 1

**Sequence alignment of TyrDC from *L. brevis* and *L. plantarum*.**

Alignment of TyrDC protein sequence from *L. plantarum* IR BL0076 and *L. brevis* strains IOEB 9809, ATCC 367 and NS77. Asterisks indicated amino acid identity.

*L. plantarum* IR BL0076 ---------MEKSNRSLKDLDLNALFIGDKAENGQLYKDLLNKLVDEHLGWRKNYIPSDP 51

*L. brevis* IOEB9809 MLNLQEVSDMEKSNRSLKDLDLNALFIGDKAENGQLYKDLLNKLVDEHLGWRKNYIPSDP 60

*L. brevis* ATCC 367 ---------MEKSNRSLKDLDLNALFIGDKAENGQLYKDLLNKLVDEHLGWRKNYIPSDP 51

*L. brevis* NS77 ---------MEKSNRSLKDLDLNALFIGDKAENGQLYKDLLNKLVDEHLGWRKNYIPSDP 51

***************************************************

*L. plantarum* IR BL0076 NMIGPEDQNSPAFKKTVGHMKTVLDQLSERIRTESVPWHSAGRYWGHMNSETLMPALLAY 111

*L. brevis* IOEB 9809 NMIGPEDQNSPAFKKTVGHMKTVLDQLSERIRTESVPWHSAGRYWGHMNSETLMPALLAY 120

*L. brevis* ATCC 367 NMIGPEDQNSPAFKKTVGHMKTVLDQLSERIRTESVPWHSAGRYWGHMNSETLMPALLAY 111

*L. brevis* NS77 NMIGPEDQNSPEFKKTVGHMKTVLDQLSERIRTESVPWHSAGRYWGHMNSETLMPALLAY 111

*********** ************************************************

*L. plantarum* IR BL0076 NYAMLWNGNNVAYESSPATSQMEEEVGQEFARLMGYDYGWGHIVADGSLANLEGLWYARN 171

*L. brevis* IOEB 9809 NYAMLWNGNNVAYESSPATSQMEEEVGQEFARLMGYDYGWGHIVADGSLANLEGLWYARN 180

*L. brevis* ATCC 367 NYAMLWNGNNVAYESSPATSQMEEEVGQEFARLMGYDYGWGHIVADGSLANLEGLWYARN 171

*L. brevis* NS77 SYAMLWNGNNVAYESSPATSQMEEEVGQEFARLMGYDYGWGHIVADGSLANLEGLWYARN 171

.***********************************************************

*L. plantarum* IR BL0076 IKSLPFAMKEVNPELVAGKSDWELLNMPTKEIMDLLENAGSQIDEVKKRSARSGKNLQRL 231

*L. brevis* IOEB 9809 IKSLPFAMKEVNPELVAGKSDWELLNMPTKEIMDLLENAGSQIDEVKKRSARSGKNLQRL 240

*L. brevis* ATCC 367 IKSLPFAMKEVNPELVAGKSDWELLNMPTKEIMDLLENAGSQIDEVKKRSARSGKNLQRL 231

*L. brevis* NS77 IKSLPFAMKEVNQELVAGKSDWELLNMPTKEIMDLLENAGSQIDEVKKRSARSGKNLQRL 231

************ ***********************************************

*L. plantarum* IR BL0076 GKWLVPQTKHYSWMKAADIIGIGLDQVVPVPIDSNYRMDIQALESIIRKYAAEKTPILGV 291

*L. brevis* IOEB 9809 GKWLVPQTKHYSWMKAADIIGIGLDQVVPVPIDSNYRMDIQALESIIRKYAAEKTPILGV 300

*L. brevis* ATCC 367 GKWLVPQTKHYSWMKAADIIGIGLDQVVPVPIDSNYRMDIQALESIIRKYAAEKTPILGV 291

*L. brevis* NS77 GKWLVPQTKHYSWMKAADIIGIGLDQVVPVPIDSNYRMDIQALERIIRKYAAEKTPILGV 291

******************************************** ***************

L. plantarum IR BL0076 VGVAGSTEEGAVDGIDKIVALRQKLQKEGIYFYLHVDAAYGGYARALFLDEDDQFIPYKN 351

L. brevis IOEB 9809 VGVAGSTEEGAVDGIDKIVALRQKLQKEGIYFYLHVDAAYGGYARALFLDEDDQFIPYKN 360

L. brevis ATCC 367 VGVAGSTEEGAVDGIDKIVALRQKLQKEGIYFYLHVDAAYGGYARALFLDEDDQFIPYKN 351

L. brevis NS77 VGVAGSTEEGAVDGIDKIVALRQKLQKEGIYFYLHVDAAYGGYARALFLDEDDQFIPYKN 351

************************************************************

*L. plantarum* IR BL0076 LQKVHAENHVFTEDKEYIKPEVYAAYKAFDQAESITIDPHKMGYVPYSAGGIVIQDIRMR 411

*L. brevis* IOEB 9809 LQKVHAENHVFTEDKEYIKPEVYAAYKAFDQAESITIDPHKMGYVPYSAGGIVIQDIRMR 420

*L. brevis* ATCC 367 LQKVHAENHVFTEDKEYIKPEVYAAYKAFDQAESITIDPHKMGYVPYSAGGIVIQDIRMR 411

*L. brevis* NS77 LQKVHAENHVFTEDKEYIKPEVYAAYKAFDQAESITIDPHKMGYVPYSAGGIVIQDIRMR 411

************************************************************

*L. plantarum* IR BL0076 DTISYFATYVFEKGADIPALLGAYILEGSKAGATAASVWAAHHTLPLNVTGYGKLEGASI 471

*L. brevis* IOEB 9809 DTISYFATYVFEKGADIPALLGAYILEGSKAGATAASVWAAHHTLPLNVTGYGKLEGASI 480

*L. brevis* ATCC 367 DTISYFATYVFEKGADIPALLGAYILEGSKAGATAASVWAAHHTLPLNVTGYGKLEGASI 471

*L. brevis* NS77 DTISYFATYVFEKGADIPALLGAYILEGSKAGATAASVWAAHHTLPLNVTGYGKLEGASI 471

************************************************************

*L. plantarum* IR BL0076 EGAHRYYDFLKNLKFEVAGKRISVHPLISPDFNMVDYVLKEDGNDDLIEMNRLNHAFYEQ 531

*L. brevis* IOEB 9809 EGAHRYYDFLKNLKFEVAGKRISVHPLISPDFNMVDYVLKEDGNDDLIEMNRLNHAFYEQ 540

*L. brevis* ATCC 367 EGAHRYYDFLKNLKFEVAGKRISVHPLISPDFNMVDYVLKEDGNDDLIEMNRLNHAFYEQ 531

*L. brevis* NS77 EGAHRYYDFLKNLKFEVAGKRISVHPLISPDFNMVDYVLKEDGNDDLIEMNRLNHAFYEQ 531

************************************************************

*L. plantarum* IR BL0076 ASYVKGSLYGKEYIVSHTDFAIPDYGDSPLAFAESLGFSEVEWRHAGKVTIIRASVMTPY 591

*L. brevis* IOEB 9809 ASYVKGSLYGKEYIVSHTDFAIPDYGDSPLAFAESLGFSEVEWRHAGKVTIIRASVMTPY 600

*L. brevis* ATCC 367 ASYVKGSLYGKEYIVSHTDFAIPDYGDSPLAFVESLGFSEVEWRHAGKVTIIRASVMTPY 591

*L. brevis* NS77 ASYVKGSLYGKEYIVSHTDFAIPDYGDSPLAFVESLGFSEAEWRHAGKVTIIRASVMTPY 591

********************************.*******.*******************

*L. plantarum* IR BL0076 MNQRENFDYFAPRIKKAIQADLEKVYASVNQKENV 626

*L. brevis* IOEB 9809 MNQRENFDYFAPRIKKAIQADLEKVYASVNQKENV 635

*L. brevis* ATCC 367 MNQRENFDYFAPRIKKAIQADLEKVYASVNQKENV 626

*L. brevis* NS77 MNQRENFDYFAPRIKKAIQADLEKVYASVNQKENV 626

***********************************
